# Ubiquitous Working: Do Work Versus Non-work Environments Affect Decision-Making and Concentration?

**DOI:** 10.3389/fpsyg.2018.00310

**Published:** 2018-03-13

**Authors:** Carolin P. Burmeister, Johannes Moskaliuk, Ulrike Cress

**Affiliations:** ^1^Knowledge Construction Lab, Leibniz-Institut für Wissensmedien, Tübingen, Germany; ^2^Wirtschaftspsychologie Department, International School of Management, Frankfurt, Germany

**Keywords:** mobile work, ubiquitous working, environmental effects, decision-making, concentration, work demands, personality

## Abstract

New communication technologies and mobile devices have enabled knowledge workers to work independently of location and in more than one fixed environment (ubiquitous working). Previous research shows that physical environments can influence cognition and work performance. We manipulated environment (i.e., a virtual office as a typical *work environment* compared to a virtual garden as a *non-work environment*) and time pressure (i.e., inducing *time pressure* vs. *no time pressure*) in order to investigate whether the environment influences decision-making and concentration. *N* = 109 students participated in this laboratory experiment. We posited (a) that a work environment would activate a work-related schema which in turn would enhance concentration performance and make decisions more risky compared to non-work environments and (b) that the environmental effect is more pronounced if time pressure is present compared to conditions where no time pressure is present. We found modest hypothesis-confirming main effects of environment on decision-making and concentration but no interaction effect with time pressure. As we used an innovative methodology that entails several limitations, future research is needed to give insights into the process and to investigate whether results hold true for all types of work settings, work demands, or work activities.

## Introduction

Due to the proliferation of handy electronic mobile devices, such as notebooks, tablets, or smartphones, people can now access data and information easily wherever they are. Mobile device systems are flexible, affordable, and easy to use (Helal et al., 2001) and they allow the economic use of mobile workspaces. New communication technologies induce new ways of working, known by different names: mobile, multi-locational, remote, flexible, distributed, or virtual work (e.g., Lönnblad and Vartiainen, 2012). Each enable employees to work in more than one fixed location. Previous research shows that it does “matter where you work” (Hill et al., 2003, p. 220; Moskaliuk et al., 2017) in the sense that the physical environment can influence cognition and work performance (e.g., Kay et al., 2004; Shalley et al., 2004; Slepian et al., 2010; Steidle and Werth, 2013; Lee et al., 2015). The design features of a workspace (e.g., lightning, furniture, acoustics, or temperature) affects well-being, work satisfaction and also work performance (e.g., Vischer, 2007, 2008). We present a laboratory experiment and discuss how the continuous change of working environments affects work behavior and performance, in terms of decision-making and concentration, depending on characteristics of the work task (e.g., whether time pressure is present or not).

For knowledge workers, one precondition for working successfully, even in multiple, different, and often non-work-related environments, is the possibility to interconnect and combine each environment, for example via some kind of cyberspace (e.g., Halford, 2005) or virtual workspace (e.g., Vartiainen and Hyrkkänen, 2010). Vartiainen and Hyrkkänen (2010) go even further and propose the idea that a mobile multi-locational worker has to work within four distinct spaces, each entailing its own resources and challenges: the physical workspace itself (e.g., home or the main workplace, trains, or cafés), virtual spaces (e.g., internet and intranet, communication tools, or knowledge platforms), social spaces (e.g., social interactions, social networks with customers, colleagues, or family members), and mental spaces (e.g., individual or shared cognitive constructs, thoughts, beliefs, or ideas). Each of these spaces influences the perception of and the behavior within the other spaces (Vartiainen and Hyrkkänen, 2010). Following these suggestions, ubiquitous, mobile workers are not only present in one physical workspace (e.g., in the main office or with a laptop in the park), but also within a combination of the other spaces. This would mean that although a worker is physically working in his living room at home (physical workspace), in his/her thoughts and current mental state s/he might be in his/her main office at the company premises (mental space). Is a successful ubiquitous worker indeed able to activate an appropriate work-related ‘mental space’ independently of his or her physical environment?

To investigate this question, theories of general cognitive processes should be considered. For example Wilson (2002) summarized several views regarding the assumption that each cognitive process occurs through an interaction with the environment. Cognition is distributed between the individual and the situation and, during a cognitive process, the information perceived within the environment affects that process. In some cases, cognition might also take place without any direct interaction with the environment (e.g., day dreaming or remembering) and these situations can be constructed with help of mental representations. In addition, schema frameworks state that the mind and knowledge of humans is organized and structured by networks of information that are activated when certain things are experienced (e.g., Mandler, 1984). Certain environments activate associated schemas, which may be characterized by knowledge, beliefs and attitudes regarding the environment, or by behavior scripts on how to act within this environment. We posit that a work-related environment activates a work-related schema which leads to an appropriate work-related mental state (e.g., being concentrated and attentive) and to appropriate work-related behavior, thus enabling high performance in work-related activities. An environment not related to work might not activate a work-related schema and therefore may lead to lower performance.

In addition Wilson (2002) states that when cognition involves time pressure, suitable processing strategies are available to guarantee fast information processing. Under pressure, there might not be enough time to generate a detailed mental model of the current environment or situation and it might be more useful to rely on representations of situations acquired through prior experiences. A large body of existing research demonstrates that time pressure affects information processing and decision-making (Kelly and Karau, 1999; Maule et al., 2000; Suri and Monroe, 2003). Incorporating these effects into our previously described assumptions, we rely on several suggestions of the heuristic-systematic model (HSM, e.g., Chaiken, 1980, 1987). The HSM was originally developed to explain information processing in persuasion but can be applied to different areas (Chaiken et al., 1989). Systematic processing is synonymous with an analytic, demanding processing style in which all relevant information and data are comprehensively processed and integrated. Therefore, several situational variables must fit: people must be motivated and sufficient resources and capacities are needed. Time can be included among these capacities and resources. If time is limited and persons experience time pressure, systematic processing becomes less likely and individuals tend to rely on heuristics in order to go easy on resources. Heuristic processing demands less cognitive effort because people focus only on limited information to formulate judgments or decisions. This limited information might include heuristics that may be activated intentionally or automatically. Heuristics are cues that might be learned through prior experiences, for example stereotypes, explicit beliefs, rules, but also schemata. Chaiken et al. (1989) propose that heuristic cues have maximal impact when motivation is low or the capacity for systematic processing is limited, for example when time is constrained. As mentioned above, we posit that environments elicit related schemas and therefore create the potential to act in terms of heuristic cues. Accordingly, the impact of such cues (e.g., activated work- vs. non-work-related schemas) on information processing should be higher when individuals experience time pressure because resources to process systematically are limited. Previous research underpins this assumption (e.g., De Dreu, 2003). Such notions help establish a suitable theoretical framework for investigating possible environmental effects on work behavior and performance (e.g., decision-making and concentration) that might emerge from ubiquitous working.

We measured work behavior and work performance of knowledge workers in terms of a *decision-making* task and a *concentration* task. The abilities to work in a highly-concentrated fashion and take risks are essential aspects of professional routines that are crucial for organizational success of occupations involving knowledge work (Ramirez and Nembhard, 2004; Reinhardt et al., 2011; e.g., managers and entrepreneurs: March and Shapira, 1987; MacCrimmon and Wehrung, 1988; Busenitz and Barney, 1997; Busenitz, 1999; Stewart and Roth, 2001; Rauch et al., 2009). There is no consensus regarding how to measure performance of knowledge workers (Ramirez and Nembhard, 2004). Therefore, we chose to investigate work behavior and work performance through two tasks that include typical activities of knowledge workers and that can be assessed in an objective, standardized way (i.e., making decisions and working with concentration).

In sum, we assume that the environment influences decision-making and concentration. We assume that work environments enhance performance in work-related activities, such as tasks that require a high amount of concentration, and make decisions riskier.

*Hypothesis 1a:* Decision-making is riskier in the work environment compared to the non-work environment.
*Hypothesis 1b:* Concentration is higher in the work environment compared to the non-work environment.

Furthermore, we assume that time pressure moderates the effect of the environment on decision-making and concentration. Under time pressure, the impact of the environment through activated work- vs. non-work-related schemata should be more pronounced and should have a stronger effect compared to conditions with no time pressure.

*Hypothesis 2a:* The enhancing effect of the work environment on decision-making and concentration performance should be more pronounced in conditions with time pressure compared to conditions with no time pressure.

## Materials and Methods

### Design

We conducted an experiment with a 2x2 between-subjects design: two *environment* conditions (work vs. non-work) and two *time pressure* conditions (no time pressure vs. time pressure), balanced for sex. Environment was manipulated with the help of virtual environments presented on a computer screen. The subjective experience of time pressure was manipulated via verbal instructions inducing time pressure or not. We measured *decision-making* and *concentration* as dependent variables with standardized tests.

### Participants

A total of 109 students volunteered to participate in the study; eight were excluded from the analysis due to technical problems. The remaining 101 participants were between 18 and 62 years old (*M* = 23.52, *SD* = 6.74), 54 were female. Participants were randomly assigned to the four experimental conditions (2 × 2, environment × time pressure). Volunteers were paid 8€ for participation or participated in exchange for course credit, and all data were recorded anonymously. This research complied with the American Psychological Association’s ethical principles and received approval from the institute’s own ethics committee.

### Procedure

**FIGURE 1:**
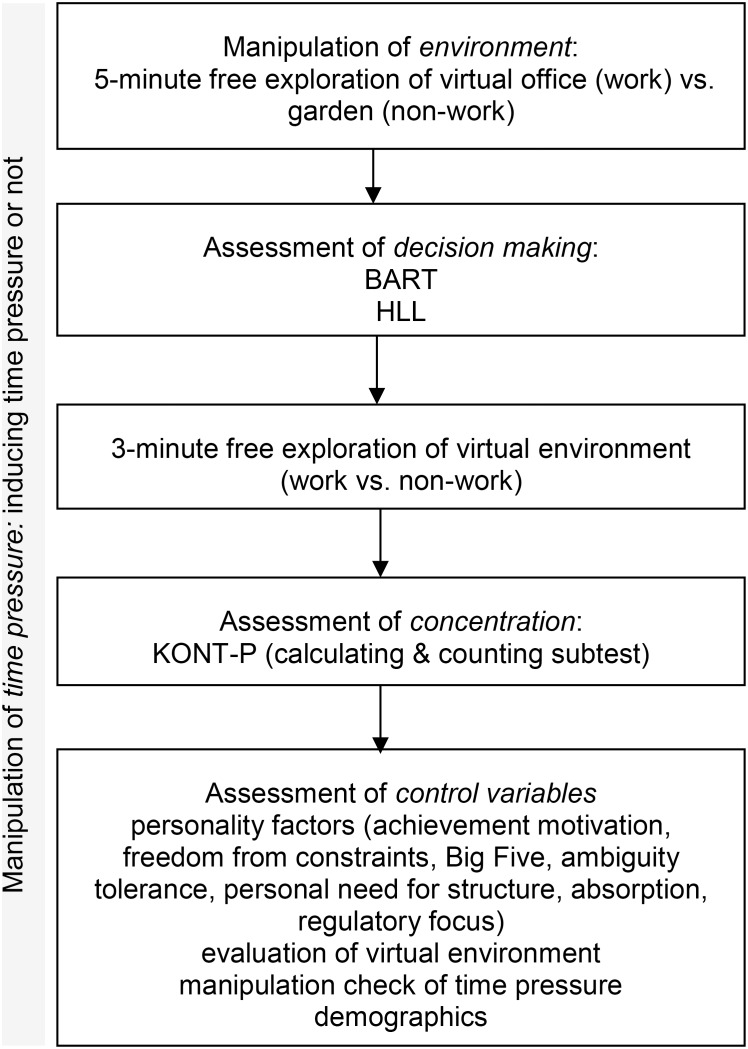
Flow chart of the procedure.

After being greeted and seated participants, signed informed-consent statements and were given written instructions. The greeting and verbal instructions differed between the no time pressure and the time pressure conditions (see section “Manipulation of Time Pressure”). Participants in both environment conditions (work vs. non-work) started the experiment with 5-min of free exploration through one of the virtual environments (virtual office or the virtual garden, see section “Manipulation of the Environment”). Participants were instructed to engage with their stay in the environment by trying to imagine themselves there in real life. Afterward, the experimental tasks and assessments began, starting with the decision-making tasks: the Balloon Analogue Reaction Task (BART) was followed by the Holy Laury Lottery (HLL). Subsequently, participants were asked to return to the virtual environment for 3 min, with the same instructions to fully immerse themselves in the environment. The assessment of variables continued with the concentration task [Psychomeda Konzentrationstest (KONT-P)], followed by questionnaires assessing control variables (achievement motivation, subjective feeling of freedom from constraints, the Big Five, ambiguity tolerance, personal need for structure, absorption capacity, and regulatory focus), and an evaluation of the virtual environment. There were also questions regarding time pressure manipulation and demographics (all assessed variables are described in Section “Measures of Work Behavior and Work Performance,” the experimental setup is described in Section “Experimental Setup”). The environment projection remained on the wall throughout the whole experiment to maintain the environmental priming (i.e., through displaying the picture section of the virtual environment, where the participant stopped in the prior exploration). All tasks and instructions were written in German. After completing the study, participants were thanked and fully debriefed. **Figure 1** depicts a flowchart of the procedure.

#### Experimental Setup

**FIGURE 2:**
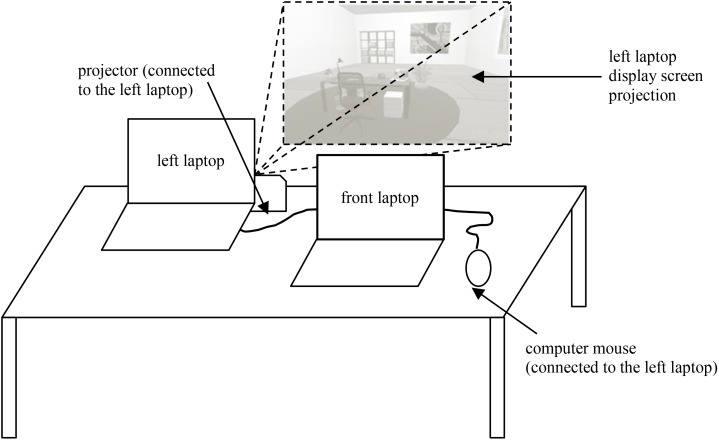
Experimental setup, each participant had his own desk, sitting in front of two laptops. A projector and a computer mouse were connected to the left laptop; the display screen of the left laptop was projected on the wall. A screenshot of the virtual office is displayed.

All participants completed the experiment with an identical experimental setup. Each participant worked on a desk with two laptop computers (15.4″ screen diagonal). One laptop computer was placed in front of the seated participant (front laptop), while the other laptop was placed slightly to the left on the desk and connected to a video projector and a computer mouse (left laptop). The assessment of the dependent variables as well as data collection was conducted on the front laptop. The participants completed all tasks and questionnaires with the help of the integrated keyboard. The presentation of the virtual environments ran on the left laptop, but the integrated monitor display was set to black and the environments were instead projected large-sized on the wall in front of the desk. Participants navigated through the environment with help of the computer mouse. **Figure 2** illustrates the experimental setup.

#### Manipulation of the Environment

**FIGURE 3:**
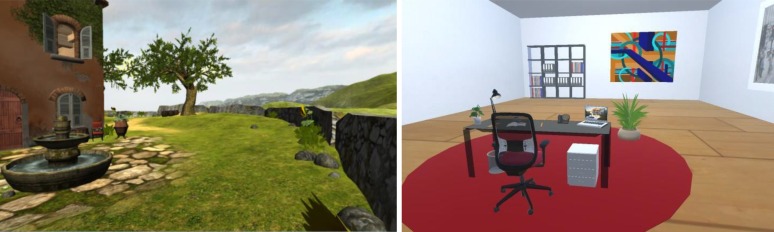
Screenshots of the virtual garden scenery with a holiday cottage, trees, and a fountain **(left)** and of the virtual office with a desk, office chair, and other office supplies **(right)**.

The environment was manipulated using two virtual environments (see also Moskaliuk et al., 2017). Virtual environment technologies are useful tools for psychological research, as they are able to create sufficiently realistic situations while being able to control for confounding variables (Blascovich et al., 2002) and have already been applied in various research areas (e.g., Cho et al., 2002; Klinger et al., 2005; Slater, 2009; Cohen-Hatton and Honey, 2015). In the work environment condition, participants navigated through a virtual office environment. The virtual office was furnished and equipped like a stereotypical office, for example, with a laptop and office supplies placed on a desk and with an office chair, a bookcase, and a potted plant in the room (see **Figure 3**, right). In the non-work environment condition, participants navigated through virtual Mediterranean garden scenery. The virtual garden included a holiday cottage (not explorable) surrounded by peaceful nature, flowers, trees, a fountain with running water, and lake view (see **Figure 3**, left).

#### Manipulation of Time Pressure

Time pressure (no time pressure vs. time pressure) was manipulated through of standardized verbal instructions in different parts of the procedure, and with the presence or non-presence of a ticking egg-timer. Participants in the *time pressure* condition were welcomed by the trained experimenter with the following standardized directions: “Unfortunately we have little time for conducting the experiment today, because the room is actually reserved for a different experiment. We will need to hurry up. I will set an egg-timer to check the time that we have.” The experimenter started the egg-timer as soon as participants sat down and began reading instructions. Participants could not see the time display of the egg-timer and had no information about when the alarm bell would start ringing (in fact, the alarm bell did not start ringing in any experimental trial). The egg-timer was ticking within earshot of the participant throughout the entire experiment. At the beginning of the second environment exploration, participants were told, “Today, we are doing this in a shorter period of time, because we don’t have as much time as usual.” After 20 min, the experimenter reminded participants verbally to hurry up. Participants in the *no time pressure* condition received verbal instructions that did not include any time constraints. Participants in the no time pressure condition were greeted with no mention of time at all, no egg-timer was started, and participants were asked if everything was going well instead of being reminded to hurry up after 20 min. In fact, participants in the no time pressure and the time pressure condition had the same amount of time to conduct the experiment. To check the manipulation, we asked “Did you experience any time pressure during the experiment?” A higher rating indicated less subjective time pressure. Participants in the no time pressure condition (*M* = 5.29, *SD* = 1.73) experienced significantly less subjective time pressure compared to participants in the time pressure condition (*M* = 4.13, *SD* = 1.96), *t*(99) = 3.12, *p* = 0.002. Therefore, the manipulation of time pressure (time pressure being present or not) can be considered successful.

### Measures of Work Behavior and Work Performance

#### Decision-Making

We used two measures of decision-making (BART and HLL) since there is still no consensus about whether decision-making is a consistent outcome or whether it depends on the way it is assessed (Kogan and Wallach, 1967).

The BART is a “computerized, laboratory-based measure” (Lejuez et al., 2002, pp 75–76) to assess *decision-making*. A small balloon, which was identical in each trial, was presented on the computer screen. Participants were instructed to press the space bar to inflate the balloon. Each press on the space bar inflated the balloon by one pump. Each balloon had a different randomized bursting point. Participants earned a hypothetical 0.10 Euros for each pump but lost all the money for a given trial if the balloon burst. Participants were instructed to press the space bar as many times as they dared to inflate the balloon. More pumps indicated a higher risk of bursting the balloon but also the possibility for a higher reward. Participants were asked to complete a total of 20 trials. The BART score was computed in terms of the total amount of money earned over all trials. A higher BART score indicated riskier decision-making.

Additionally, we used the HLL (Holt and Laury, 2002) as another measure of *decision-making*. The HLL measures decision-making in the financial domain and consists of 10 independent, randomly presented decision trials. In each trial, participants were asked to decide between two stock options that represented more or less risk. The more risky option was defined as a lower chance of winning a higher amount of money (e.g., winning 385 Euros with a probability of 10% and 10 Euros with a probability of 90%), the less risky option was defined as a higher chance of winning a smaller amount of money (e.g., winning 200 Euros with a probability of 10% and 160 Euros with a probability of 90%). Participants had to weigh the chances of winning and the possible amount of money to be gained, and then make either the risky or the less risky decision. We assigned a score of 1 if participants chose the less risky option and a score of 2 if participants chose the riskier option. An overall risk score for all decisions was summed as a measure of decision-making (HLL Score), with a higher score indicating riskier decision-making. One of the items in the lottery served as a control for careful processing of the task as choosing option 1 indicates careless clicking through the task (option 1: winning 200€ with a probability of 100% and 160€ with a probability of 0%; option 2: winning 385€ with a probability of 100% and 10% with a probability of 0%). Participants who did not work carefully were excluded from the analysis of the HLL, and eight participants (*n* = 93) were thus excluded.

#### Concentration

The KONT-P by Satow (2011) was used to measure *concentration* capability in terms of quantity (accuracy and speed) and quality (efficiency). The KONT-P consists of one calculating and one counting subtest. The calculating subtest consisted of simple addition tasks. In the counting subtest, participants had to count the number of times the digit 1 appeared in a row of distracting digits and letters. For each subtest, five pages were presented successively, with seven tasks at a time. Participants were asked to solve as many tasks in 20 s as possible. After 20 s, the next page was forwarded automatically. Participants were told that it was not possible to solve all of the tasks within the restricted time. The KONT-P offers several measures for concentration capability (Satow, 2011): *accuracy, speed, efficiency, accuracy increase*, and *speed increase. Accuracy* was defined as the sum of correctly solved tasks; *speed* was defined as the sum of completed tasks. Accuracy and speed were calculated for both subtests as well as for the overall test. *Efficiency* was defined as the ratio between accuracy and speed in the overall test. In addition, *accuracy increase* and *speed increase* throughout the entire test was measured in terms of the difference between accuracy or, respectively, speed in the first half of the test compared to the second half.

### Control Variables

To control for confounding effects, we assessed several control variables at the end of the study. As personality has been shown to influence a variety of work outcomes (e.g., Humphreys and Revelle, 1984; Van Yperen et al., 2014) we included several personality factors as control variables that have either been found to affect concentration and decision-making or that can be assumed to interact with the environment or with time pressure. Unless otherwise noted, all items of the personality questionnaires were rated on a five-point Likert scale. *Achievement motivation* was assessed with 10-items derived from the Leistungsmotivationsinventar (LMI-K; Schuler et al., 2001). Higher values indicated higher achievement motivation. *Freedom from constraints* was assessed, as suggested by Steidle and Werth (2013), in terms of subjectively perceived self-regulation (externally controlled vs. self-determined, see Ryan and Deci, 2000) and assertiveness (inhibited vs. self-assured, see Jacobs and Scholl, 2005). Higher values indicated a more autonomous self-regulation. Personality traits in terms of the *Big Five* (openness to experience, conscientiousness, extraversion, agreeableness, neuroticism) were assessed using the 10-item Big Five Inventory (BFI-10; Rammstedt et al., 2013). Higher values indicated a more pronounced manifestation of the trait. *Ambiguity tolerance* was assessed with the Ungewissheitstoleranzskala (UGT) by Dalbert (1999) including eight-items. Higher values indicated greater tolerance of ambiguous situations. *Personal need for structure* was measured with 12-items of the personal need for structure scale (PNS) by Machunsky and Meiser (2006). Higher values indicated a stronger personal need for structure. *Regulatory focus* was measured with the 10-item Regulatory Focus Scale (RFS) by Fellner et al. (2007). Higher values indicated a more promotion-oriented regulatory focus; lower values indicate a more prevention-oriented regulatory focus. *Absorption capacity* was measured with the help of eight-items derived from the Expanded Tellegen Absorption Scale (ETAS) by Smith-Jackson and Klein (1997). Higher values indicated a higher capacity for absorption.

In addition we assessed *evaluation of virtual environment* by means of several questions regarding the virtual reality experience. One-item was implemented to rate *dizziness and nausea* due to the 3D presentation of the virtual environments, higher values indicated stronger physical discomfort. *Immersion* was assessed by five-items derived from the presence questionnaire by Witmer and Singer (1998). Higher values indicated a stronger immersion in the virtual environment. Additionally, three questions regarding *pleasure* (“How much fun have you experienced exploring the 3D environment?,” 1 = no fun at all; 7 = a lot of fun), *motivation* (“How motivated have you been during the exploration of the 3D environment?,” 1 = no motivation at all; 7 = a lot of motivation), and *feeling* (“How did you feel during the exploration of the 3D environment?,” 1 = very bad; 7 = very good) were asked to control for effects of the environment. All items were rated on a **seven**-point Likert scale. Higher values indicated more desirable conditions. Demographic variables of interest were gender and age.

## Results

*t*-Tests for independent samples were computed to investigate differences between two groups. Interaction effects were assessed using univariate variance analyses. Moderation analyses were conducted with the help of PROCESS modeling, as recommended by Hayes (2012). There were no indications of outliers.

### Hypotheses 1a and 1b

First, we examined whether there were any simple main effects of the environment on decision-making (Hypothesis 1a) and concentration (Hypothesis 1b), without considering time pressure. We found a marginally significant main effect of the environment on *decision-making* as measured with the HLL, and a significant main effect on *accuracy increase* in the KONT-P. Participants in the work environment (*M* = 14.19, *SD* = 1.62) showed marginally significantly riskier *decision-making* behavior compared to participants in the non-work environment (*M* = 13.65, *SD* = 1.42), *F*(1,92) = 2.94, *p* = 0.090. Further, participants in the work environment (*M* = 3.60, *SD* = 3.06) showed a significantly higher *accuracy increase* compared to participants in the non-work environment (*M* = 2.24, *SD* = 2.68), *F*(1,100) = 5.53, *p* = 0.021. We did not find any other main effects of the environment on decision-making or concentration performance (all other *p* > 0.150).

### Hypothesis 2

We assumed that the effects of the environment on decision-making and concentration are moderated by time pressure (Hypothesis 2). We did not find such an interaction between the environment and time pressure (all *p* > 0.264).

We examined whether time pressure alone showed any effects on decision-making and concentration performance. We found two significant main effects of time pressure, and these were on *accuracy* and *speed* in the calculating subtest. Participants in the no time pressure condition (*M* = 18.61, *SD* = 3.91) were *more accurate* compared to participants in the time pressure condition (*M* = 17.17, *SD* = 3.64), *F*(1,100) = 4.07, *p* = 0.046. Further, participants in the no time pressure condition (*M* = 20.12, *SD* = 3.94) were *slower* compared to participants in the time pressure condition (*M* = 18.69, *SD* = 3.52), *F*(1,100) = 4.13, *p* = 0.045. We did not find any other main effect of time pressure on decision-making and concentration performance (all other *p* > 0.112).

### Control Variables

#### Personality Factors

We did not find any *a priori* differences between participants in the work and non-work condition neither for achievement motivation (*p* = 0.810), nor for freedom from constraints (*p* = 0.350), the Big Five (all *p* > 0.113), ambiguity tolerance (*p* = 0.309), personal need for structure (*p* = 0.415), regulatory focus (*p* = 0.496), or absorption capacity (*p* = 0.099).

#### Evaluation of Virtual Environment

The evocation of nausea due to the 3D-presentation of environments differed with marginal significance between both environments. The work environment (*M* = 6.94, *SD* = 0.42) evoked with marginal significance less nausea compared to the non-work environment (*M* = 6.57, *SD* = 1.34), *t*(56.69) = 1.86, *p* = 0.069. As the mean values for both environments are not very high (note: scale was 1 to 7, with 7 indicating no nausea at all), this result was not a cause for concern. The subjective feeling of immersion did not differ significantly between the work environment (*M* = 4.90, *SD* = 0.99) and the non-work environment (*M* = 5.16, *SD* = 1.05), *t*(99) = -1.28, *p* = 0.205. Thus, environments were constructed in a comparable manner. Regarding pleasure, motivation, and feeling elicited through the virtual environment, the two environments differed significantly. Participants experienced significantly less pleasure in the work environment (*M* = 4.25, *SD* = 1.56) compared to participants in the non-work environment (*M* = 5.16, *SD* = 1.30), *t*(99) = -3.19, *p* = 0.002. Participants also had less motivation to experience the work environment with marginal significance (*M* = 5.12, *SD* = 1.58) compared to participants in the non-work environment (*M* = 5.65, *SD* = 1.20), *t*(94.80) = -1.93, *p* = 0.056. Additionally, participants felt significantly better while experiencing the non-work environment (*M* = 5.57, *SD* = 1.34) compared to the work environment (*M* = 4.94, *SD* = 1.23), *t*(99) = -2.46, *p* = 0.015. We ran several mediation analyses to make sure that these environment-associated variables did not mediate effects of the environment. We did not find any mediation effects of nausea (all *p* > 0.250), immersion (all *p* > 0.416), pleasure (all *p* > 0.216), motivation (all *p* > 0.212), or feeling (all *p* > 0.410), for any of the dependent variables.

## Discussion

### Summary

This study investigated whether the environment affects decision-making and concentration performance moderated by demands of the task, such as time pressure. We manipulated *environment* (work vs. non-work) and *time pressure* (working without vs. with time constraints) to investigate influential effects on decision-making or concentration.

We assumed that a work environment is associated with a work-related schema that would in turn activate cognitive resources and associations related to work behavior (e.g., working with high concentration and daring more), which then in turn would enhance concentration performance or affect decision-making behavior. We found a significant main effect of environment on concentration regarding accuracy increase in the concentration task. As expected, participants in the work environment showed a higher accuracy increase compared to participants in the non-work environment. In addition, we found a marginally significant effect of environment on decision-making when measured with the Holt Laury Lottery. Participants in the work environment made marginally more risky decisions compared to participants in the non-work environment.

In addition, we assumed that time pressure strengthens the effect of the environment on decision-making behavior and concentration performance, as the impact of heuristic cues (e.g., schemata activated by the environment) is stronger when resources (e.g., time) are limited. Time pressure (and therefore experienced stress) is a common challenge in work life (e.g., Blaug et al., 2007; Perlow, 2016) and has been shown to have an impact on cognitive processes and performance (e.g., Lazarus et al., 1952; Mendl, 1999). Time pressure affects information search strategies and decision-making (e.g., Wright, 1974; Ben Zur and Breznitz, 1981; Huber and Kunz, 2007) as well as processing strategies (e.g., Verplanken, 1993; Suri and Monroe, 2003) and performance in general (Andrews and Farris, 1972). However, results in this experiment did not reveal a significant interaction effect between environment and time pressure for any of the dependent variables but two significant main effects emerged for time pressure on accuracy and speed in one of the subtests of the concentration task. Participants who did not experience time pressure worked more accurately and showed slower task processing. This result is consistent with our manipulation check of the time pressure induction, which showed that participants who experienced time pressure did in fact work faster compared to participants who did not experience time pressure. It seems obvious that accuracy might suffer when task processing speed is higher because participants either do not have the time or do not take the time to check each task or trial carefully.

### Limitations

By manipulating the environment with the short exploration of a virtual reality, we found effects on subsequent decision-making and concentration performance. Although the effect sizes are modest, these findings provide initial evidence and suggest that the effects of real environments might be stronger.

Several limitations of the study might have dampened the effects, either due to weak points in theory or in the experimental design. Results were not strong enough to confirm the hypothesis that work environments enhance performance in concentration and make decisions riskier compared to non-work environments. On the one hand, it might be possible that we were not able to activate the intended mental schema or that we accidentally caused competing effects besides the ones we intended by means of the environment manipulation. We compared a typical work environment (a traditional office) to only one potential representative of a non-work environment (garden scenery). This brings at least two difficulties. First, we cannot be sure that the presented environments (work vs. non-work) hold equally strong associations with the intended mental schemas (work vs. non-work) we planned to activate by it. Whereas a traditional office might be typical enough to hold associations with work for almost everybody, we do not know whether peaceful garden scenery is associated with a typical non-work environment with the same strength. The presented garden resembles a Tuscan landscape and might not be familiar to every participant. Therefore, we need to consider the possibility that the non-work environment did not activate a sufficiently strong non-work schema for everybody. Second, a large body of research showed that natural environments have benefits on health and well-being as well as cognition through attention restoration (e.g., Largo-Wight et al., 2011; Richardson et al., 2016). For example, Berman et al. (2008) compared cognitive functioning of participants after interacting either with natural or urban environments by means of an actual walk within the environment or by viewing pictures of the environment. Berman et al. (2008) showed that an interaction with natural environments but not urban environments led to improved performance in executive and directed-attention attention tasks. In the Attention Restoration Theory (Kaplan, 1993, 1995) it is stated that natural environments can have restorative effects when directed attention is depleted and information processing might therefore be impaired. It is assumed that natural settings contain modestly captivating stimuli (e.g., a nice flower in the grass) that grab attention during bottom–up processes, while directed attention resources (top–down processes) can be restocked. This should lead to improved performance for subsequent tasks that require high attention. In contrast, urban settings contain acutely attention-grabbing stimuli (e.g., watch where you go and take care of the traffic) that impedes attentional resources to be restocked (Kaplan, 1993, 1995). A large body of research demonstrates these restorative, stress-reducing effects of natural environments (e.g., see review by Korpela et al., 2014). The virtual environments we used to manipulate surroundings to activate related schemas were indeed not comparable regarding natural representations. Whereas the office environment did only contain a small pot plant, the garden scenery consisted almost exclusively of natural elements such as trees, grass, flowers, or a lake. Therefore, we must consider the possibility that the natural stimuli in the non-work environment (garden scenery) did enable restoration effect on cognitive processes which the work environment did not. This restoration effect might have canceled out the intended effects of the non-work schema (e.g., showing less effort in work related activities) and led to a disconfirmation of our initial hypotheses. Future research must control for these potential confounding effects by using different non-work environments in contrast with the work environment. Possible suitable non-work environments should include as many natural elements as the work environment. For example, a room with the same size and architecture as the work-related office room but instead furnished with typical non-work-related items such as a sofa, a TV, or gaming consoles. However, when we designed the reported experiment, we decided to use the garden scenery as a non-work environment as a starting point for this highly complex research area. The goal of future research studies is to refine the design (e.g., through using different gradients of differences between the environments) in order to exclude possible confounders and identify the relevant factors. Furthermore, we found several differences between the work and non-work environments regarding the control variables motivation, pleasure, and feeling. Participants exploring the non-work environment felt better, indicated more pleasure and higher motivation. Although we did not find mediating effects of these control variables, this should be kept in mind when designing comparable work/non-work environments for future research.

In addition, the manipulation of environments might not have been strong enough to elicit sufficient related schemas because of the rather short manipulation time or the characteristics of the laboratory where the experiment took place. The duration of the virtual environment exploration was 5 min the first time and 3 min the second time, which might not have been long enough to activate a strong mental schema. Moreover, participants might have had difficulties to maintain the activated mental schema throughout the course of the experiment. To avoid the extinction of the schema, we tried to keep duration of tests after the exploration as short as possible and repeated the environment manipulation. After conducting the decision-making tasks, a second environment exploration followed which functioned as a mental break to prevent carry-over effects from the decision-making to the concentration task. We randomized items within all tasks, but unfortunately it was not possible to randomize sequence of decision-making and concentration tasks due to the technical restrictions of the BART. Special software is needed to conduct the BART and it was not possible to directly include it in the same survey software as the other measurements as it had to be started manually. However, we cannot make sure that these actions (e.g., short test duration and repeated environment manipulation) were enough to maintain the intended mental schemas throughout the assessment of dependent variables. Participants still sat in the laboratory throughout the experiment, which might have contained additional stimuli that might have counteracted the intended effects. The laboratory resembled a traditional office (and therefore a work environment) in a much stronger way than it resembled a non-work environment, such as a garden. If participants have lifted their heads after exploring the virtual environments or at some point during the experimental course, elements of the laboratory (e.g., desks, computers, work utensils) might have grabbed their attention. These elements might have elicited a concurring mental schema, which in turn might have suppressed especially the activation of a non-work-related schema.

### Implications for Future Research

We cannot draw sufficient conclusions from our experiment to support or to contradict these speculations. We assumed that environments activate associated mental schemas but within the current experimental design we were not able to directly measure these mental schemas. We first tried to investigate them in a way that we examined the effects of the mental schema on performance and behavior. As we did not find robust effects, we cannot differentiate whether this lack is due to unsuccessful manipulation (activated mental schemas were not activated in a sufficient way), due to an insufficient theoretical base (environments do not activate related schemas), or whether hypotheses (environments have the potential to affect decision-making and concentration) must be rejected. Future research is needed to unravel these factors and it will be necessary to distinguish the physical from the mental environment (and the schema activated by each) to identify the underlying process. Up to now there is no sound method to measure mental schemas directly. However, one possible way might be to add another method of manipulation to the experimental design: to manipulate work- and non-work environments not only in fact (by means of virtual environments or actual environments) but also by means of priming methods (priming a work-related vs. non-work-related environments). Findings could help to understand, whether effects on performance and behavior might come from the actual environment itself or also from the mere thinking of an environment (and therefore from a mental schema).

In addition, there are several factors that might play an additional role in the relationship between environment and work performance or behavior. For example, work performance is linked to many other factors, such as job satisfaction and organizational commitment (e.g., Shore and Martin, 1989), organizational climate (e.g., Pritchard and Karasick, 1973), opportunities (e.g., Blumberg and Pringle, 1982), as well as individual differences. In everyday life, most of us know examples of successful workers who can block out every noise to work in an atypical environment, for example even on a muggy train or in a crowded café. However, there are also contrary examples of workers who need a tranquil and organized setting to work successfully. Therefore, it seems useful to consider individual characteristics to explain why some people can work under unfavorable conditions and others not. Individual differences should be kept in mind when investigating the influence of the physical environment on work outcomes. Some research has already identified a lengthy list of individual differences that have been shown to affect cognitive processes or decision-making, such as affect (e.g., Clore et al., 2001) or differences in neural correlates (e.g., Pennington, 1994) and individual risk propensity, impulsivity, or sensation seeking (e.g., Zuckerman and Kuhlman, 2000; Lejuez et al., 2002; Nicholson et al., 2005). From a practical perspective, it is crucial to investigate whether there might be individual differences in the capacities of knowledge workers to maintain work performance on an equally satisfying level in different environments and if there are certain personality factors that go into making good, successful workers.

Another important point to discuss is the method we chose to measure work outcomes. Due to practicability and reasonableness, we had to narrow down the large field of work performance and work behavior to only two basic work activities: decision-making and concentration assessed by three tasks. This is of course not enough to give an exhaustive insight in work outcomes. Vischer (2008) criticizes that in workspace research, work performance or work productivity is mostly measured in terms of self-reports. As these might be biased, it is important to use objective indicators as well. We wanted to address this concern and chose to measure decision-making and concentration by means of highly objective, standardized, hard measures and therefore our experiment is one approach to get more holistic insights into the environment–work outcome relationship. However, this poses two challenges. First, it is possible that our measurements have made it especially difficult to find an effect. Effects might not have been strong enough to influence these robust assessments. Results might have looked different if we would have included soft measures, such as self-reports, as well. Second, narrowing down real work outcomes to standardized objective tests involves a reduction in proximity to everyday work life. A standardized concentration task might not exactly reflect typical work tasks that involve high concentration. This holds true for the decision-making tasks as well. Decision-making has been shown to be multidimensional and knowledge workers are confronted daily with multiple decisions from various domains that also depend highly on situational variables. That is why it is very difficult to assess this work behavior in the limitations of only two different tasks (e.g., the BART and the lottery). Extended research is needed to be able to draw conclusions from basic task components and to transfer it to actual work behavior.

At the onset, research in innovative and rarely studied topics necessitate that not all the factors are clear and that changes in the design and method are necessary. The goal of this study was to establish the first insights into the question of whether modalities of modern work such as ubiquitous working (i.e., working in multiple locations) have effects on work performance and work behavior. This experiment was only the first step toward understanding this highly complex subject matter. Future research is needed to understand the process (e.g., whether environments elicit related mental schemas) and to investigate whether results hold true for all types of work settings (besides typical offices vs. garden sceneries), work activities (besides decision-making and concentration), different demands or characteristics of the task (besides time pressure) and for individual personalities.

## Ethics Statement

This study was carried out in accordance with the recommendations of the American Psychological Association’s ethical principles with written informed consent from all subjects. All subjects gave written informed consent in accordance with the American Psychological Association’s ethical principles. The protocol was approved by the Leibniz-Institut für Wissensmedien (IWM) own ethics committee.

## Author Contributions

All persons who meet authorship criteria are listed as authors, and all authors certify that they have participated sufficiently in the work to take public responsibility for the content, including participation in the concept, design, analysis, writing, or revision of the manuscript.

## Conflict of Interest Statement

The authors declare that the research was conducted in the absence of any commercial or financial relationships that could be construed as a potential conflict of interest.
